# Risk-based eradication as a control measure to limit the spread of LA-MRSA among Danish pig herds – a simulation study

**DOI:** 10.1038/s41598-019-49752-3

**Published:** 2019-09-13

**Authors:** Jana Schulz, Anette Boklund, Nils Toft, Tariq Halasa

**Affiliations:** 1 Technical University of Denmark, National Veterinary Institute, Kemitorvet, Building 204, 2800, Kgs. Lyngby, Denmark; 2 Federal Research Institute for Animal Health, Friedrich-Loeffler-Institut, Institute of Novel and Emerging Infectious Diseases, Südufer 10, 17493 Greifswald, Insel Riems Germany; 3 University of Copenhagen, Faculty of Health Sciences, Grønnegårdsvej 8, 1870 Frederiksberg C, Denmark

**Keywords:** Risk factors, Diseases, Mathematics and computing

## Abstract

A national screening in 2016 identified 88% of Danish pig herds positive for livestock-associated Methicillin-resistant *Staphylococcus aureus* (LA-MRSA). This highlights the importance of evaluating potential control measures that could reduce the prevalence of LA-MRSA among Danish pig herds. In addition to describing the effects of (1) reduced within-herd transmission, (2) increased biosecurity, and (3) movement restrictions, the eradication of LA-MRSA as a potential control measure was investigated using a simulation model mimicking the spread of LA-MRSA among pig herds between 2006 and 2015. The latter strategy was simulated either as eradication of a random selection of herds for surveillance or as a risk-based selection of herds based on their potential to spread LA-MRSA via pig movements in four different scenarios: low- vs. high-prevalence scenarios with control measures starting in 2007 and in 2010. Almost all control measures showed the potential to reduce the spread of LA-MRSA among pig herds, especially when implemented intensively and when control measures were combined. Risk-based selection of herds for eradication led to a greater relative reduction compared to random selection. In the high-prevalence scenario in particular, combinations including risk-based eradication led to the greatest relative reduction.

## Introduction

Within the pig population, livestock-associated Methicillin-resistant *Staphylococcus aureus* (LA-MRSA) is an opportunistic pathogen that does not usually cause clinical signs and thus no treatment is required. However, transmission from pigs to humans can occur^1^. Under unfavourable conditions, LA-MRSA can cause severe infections that might be hard to treat in humans because LA-MRSA is resistant to a wide range of antibiotics. To limit the risk of LA-MRSA spreading from pigs to humans, an effective strategy to control LA-MRSA within pig herds is needed.

The prevalence of LA-MRSA-positive herds in Denmark has increased dramatically since 2006, reaching 88% in 2016^2^. A national action plan was therefore established in 2015 to control LA-MRSA in pig herds. Among other control measures, the plan aims to reduce the use of antibiotics by 15%^3^. However, such a reduction might not be sufficient as recent research results have shown that drastic reductions in antibiotic use are needed to limit LA-MRSA spread^4,5^. These reductions might not be realistic in industrialised farms, and other measures must therefore be sought.

A simulation model mimicking the spread and control of LA-MRSA among Danish pig herds between 2006 and 2015 investigated the effects of the following control measures and combinations thereof on LA-MRSA spread among pig herds^5,6^: (1) a reduction in herds using high-risk antibiotics such as tetracyclines and β-lactams, i.e. modelling herds with reduced within-herd prevalence, (2) increased biosecurity, i.e. assuming a reduced probability of indirect LA-MRSA transmission via humans, (3) movement restrictions, i.e. prohibiting the movement of pigs from LA-MRSA-positive to LA-MRSA-negative pig herds, and (4) eradication of LA-MRSA in 5–7.5% of the herds tested positive for LA-MRSA. Only intensive control programmes that combined the four control options showed good potential for limiting the spread of LA-MRSA among pig herds^5^. However, the observed prevalence in Denmark in 2016 was substantially higher than the scenarios investigated by Schulz *et al*.^5^. It is therefore important to evaluate the success of these measures under a high initial prevalence, and to study the effect of other control options that may aid in the control of LA-MRSA in Danish pig herds.

Pig movements were shown to be a driving factor in the spread of LA-MRSA^6–9^. Based on herd-specific characteristics, herds could be categorised by their potential to spread a pathogen via pig movements. The size of the out-going contact chain is defined as the number of herds that could potentially receive pigs from one specific herd either (1) directly via pig movements from another herd, or (2) indirectly via other pig herds, within a certain time period and taking into account the temporal order of pig movements^10^. It could be used to identify herds with a greater potential to spread a pathogen via pig movements due to the higher number of contacts^11,12^. An eradication process focusing on these herds might prove useful, given the unrealistically high economic losses associated with eradicating the bacteria from all positive herds with the current high prevalence^13^.

Using the original model by Schulz *et al*.^5,6^, this study aims to evaluate the effect of risk-based eradication, i.e. in herds with a greater potential to spread LA-MRSA (hereafter referred to as hubs for LA-MRSA spread). Additionally, we investigated (1) the influence of the initial between-herd prevalence (at national level) when initiating a control programme (low-prevalence vs. high-prevalence scenario), and (2) the effects of a later initiation (2007 vs. 2010) in both the low- and high-prevalence scenarios on the potential of each individual control measure and their combinations to reduce the spread of LA-MRSA among pig herds.

## Materials and Methods

The following two chapters briefly explain the original model and describe in detail the enhancements made to carry out the presented study. All processes and parameters were described in detail in Schulz *et al*.^6^ and Schulz *et al*.^5^. Each model run was repeated 250 times, which was deemed sufficient for the model to converge and cover the variability in the simulated scenarios. The model was programmed in R version 3.2.2. - “Fire Safety”^14^.

### Simulation model

Herd information from 12,874 active Danish pig herds and 993,474 registered pig movements among these herds were used in an agent-based simulation model mimicking the spread and control of LA-MRSA among Danish pig herds between January 2006 and December 2015^5,6^. For each herd, a unique identification number, the herd type (breeding and multiplier herd, production herd, weaner herd, organic pig herd or hobby herd), and the number of registered sows, weaners and finishers were available. Herd types and herd sizes were assumed to be constant during a considered simulation year and were updated on each 1^st^ January according to the initial data set.

In the original model, within-herd spread was modelled as a three-compartment SIS model (sow-, weaner-, and finisher-compartment). Transmission between the compartments was modelled mimicking the high-risk transmission routes following the production line (e.g. from the sow to weaner and from the weaner to finisher compartments) as well as low-risk transmission routes to mimic the spread of LA-MRSA not related to the production process (e.g. via contaminated equipment). Thus, a set of three transmission parameters was used for each herd, where higher transmission rates were assumed for herds using high-risk antimicrobials such as tetracyclines and β-lactams^6^.

To mimic the between-herd spread, transmission was modelled via pig movements or via indirect contact among pig herds. Pig movements were registered in the Central Husbandry Register (CHR) and consisted of the date of the movement, the number of pigs moved (batch size), and the unique identification numbers of the sending and receiving herds. If the sender was LA-MRSA negative, no pig movement was modelled as transmission was not possible. If the sending herd was LA-MRSA positive, the number of positive pigs in the movement batch was estimated based on the within-herd prevalence. The number of LA-MRSA-positive pigs in the receiving herd was updated and thus an increased prevalence was simulated.

Indirect transmission among pig herds was modelled via two routes: (1) humans visiting more than one pig herd per day, and (2) trucks collecting pigs for slaughter. The number of indirect contacts was calculated based on a Poisson distribution. As no data were available for humans visiting pig herds, the mean (lambda) for the Poisson distribution was estimated as described elsewhere^5,15^. Data on the collection of pigs sent for slaughter were available in the movement register and used to calculate a herd-specific lambda for abattoir movements. The probability of transmission via both humans and trucks was modelled as PERT distributions based on expert opinions^6^.

Two default scenarios were established: for the low-prevalence scenario, LA-MRSA was introduced by random selection of 400 production herds and 10 breeding and multiplier herds on 1^st^ January 2006 and again on 1^st^ January 2009^6^. In the high-prevalence scenario, 10,000 herds were selected at random and independent of the registered herd type to be LA-MRSA positive on 26^th^ December 2006.

### Control strategies

Four control measures were implemented in the original study^5^: (1) reduced within-herd spread of LA-MRSA in 50% or 100% of the herds, mimicking the termination of use of high-risk antibiotics in these herds (Scenario acronyms: AB (50%) and AB (100%)), (2) reduced probability of indirect transmission of LA-MRSA via humans by 50% or 75%, mimicking increased biosecurity in all herds (Scenario acronyms: ProbIT (50%) and ProbIT (75%)), (3) movement restrictions prohibiting the movement of pigs from LA-MRSA-positive to LA-MRSA-negative herds based on testing all herds once or four times per year (Scenario acronyms: MR (1/year) and MR (4/year)), and (4) eradication of LA-MRSA (i.e. sending pigs for slaughter, cleaning and disinfecting the facilities and re-stocking with LA-MRSA-negative pigs) in randomly selected herds tested positive for LA-MRSA (Scenario acronym: EradR).

In the random eradication process, 7.5% of the breeding and multiplier herds and 5% of all other herd types were randomly selected to initiate the eradication process after testing positive for LA-MRSA in the simulation. During the eradication process, no pig movements were simulated. If pig movements were registered during the eradication period, these movements were ignored. The duration of the eradication was dependent on the herd type and number of animals within the herd^4^.

To model a risk-based eradication process, we enhanced the original data set by adding the size of the out-going contact chain, which involved counting the number of herds that received pigs from the respective herd directly or indirectly via pig movements within the considered year. When LA-MRSA screening was simulated, positive herds were arranged in order according to the size of their out-going contact chain, and 7.5% of the breeding and multiplier herds and 5% of the other herd types with the highest values of out-going contact chain were chosen for initiating the eradication process (Scenario acronym: EradH).

All control measures were initiated individually and combined in all possible combinations. To enable comparison between two scenarios, the relative reduction was calculated as the proportion *R*_*s*_ for each scenario *s*, as follows:

$${R}_{s}=\frac{\overline{Pre{v}_{d}}-{\overline{Prev}}_{s}}{{\overline{Prev}}_{d}}$$

where ${\overline{{\rm{Prev}}}}_{{\rm{s}}}$ is the predicted median prevalence of scenario s and ${\overline{{\rm{Prev}}}}_{{\rm{d}}}$ is the predicted median prevalence of the default scenario. The effects of control measures were measured six years after the initiation of control to enable comparison between the different scenarios.

## Results

### Effects of an early initiation of control

A low- and a high-prevalence scenario were set up in this study to allow evaluation of their influence on the effects of control measures. Table 1 shows the predicted prevalence in both scenarios on 1^st^ January 2007 and 1^st^ January 2010 (on the potential start day of control). We observed an increase in the predicted median herd prevalence in the low-prevalence scenario between 2007 and 2010. In contrast, in the high-prevalence scenario, the predicted median herd prevalence decreased slightly, even without active control measures (Table 1).

**Table 1:** Predicted median herd prevalence at the start of the control measures for early and late initiation and in the low- and high-prevalence scenarios.

Scenario description	Date of prevalence estimate	Low-prevalence scenario: Predicted median herd prevalence (in %) [90% prediction interval]	High-prevalence scenario: Predicted median herd prevalence (in %) [90% prediction interval]
Early initiation	1^st^ January 2007	3 [3–5]	67 [66–68]
Late initiation	1^st^ January 2010	20 [17–25]	62 [60–63]

To allow for comparison between the low- and high-risk scenarios, previously published results of the low-prevalence scenario were included to this manuscript, again. Control measures were initiated on 1^st^ January 2007 in both the low- and high-risk scenarios. When the measures were implemented individually, a reduction in the use of high-risk antibiotics in all herds led to a relative reduction of 38% in the predicted herd prevalence in the low-prevalence scenario (Scenario 1.2, Table 2). In the high-prevalence scenario, eradication of hubs led to the greatest reduction (18%) in predicted herd prevalence (Scenario 1.8, Table 2). Two control measures did not lead to any reduction in the low-prevalence scenario: a reduction in high-risk antimicrobials in 50% of the herds (Scenario 1.1, Table 2) and movement restrictions based on testing all herds once per year (Scenario 1.5, Table 2). Reducing the probability of transmission related to indirect contact by 75% as an indicator of increased levels of biosecurity (Scenario 1.4, Table 2) led to the second greatest reduction rate in the low-prevalence scenario. In contrast, in the high-prevalence scenario, increased levels of biosecurity led to a worse performance independent of the chosen reduction proportion (Table 2). In general, the relative reduction rates observed in the high-prevalence scenario were smaller than the obtained reductions in the low-prevalence scenario, except for the two scenarios where no reduction was observed in the low-prevalence scenario. The risk-based eradication process clearly led to greater reduction rates compared to the random eradication process in both the low- and high-prevalence scenarios.

**Table 2:** Predicted median herd prevalence of the default scenarios run without any control measures (first row) and relative reductions (in brackets) observed for the individual control measures six years after initiation of control (early initiation: predictions on 31^st^ December 2012, late initiation: predictions on 31^st^ December 2015).

Scenario ID	Scenario acronym	Scenario description	Predicted median herd prevalence in %* [90% prediction interval] (relative reduction)			
			Early initiation of control		Late initiation of control	
			Low-prevalence scenario	High-prevalence scenario	Low-prevalence scenario	High-prevalence scenario
0	**No control**		47 [42–52]	69 [67–71]	62 [59–65]	71 [69–73] (0%)
1.1	AB (50%)	Termination of use of high-risk antibiotics in 50% of the herds	*47* [*30*–*60*] *(0%)*	65 [55–74] (5%)	59 [46–70] (6%)	66 [60–75] (7%)
1.2	AB (100%)	Termination of use of high-risk antibiotics in 100% of the herds	**29 [13–44] (38%)**	60 [38–69] (13%)	**48 [26–60] (24%)**	61 [41–74] (14%)
1.3	ProbIT (50%)	Probability of indirect transmission via humans reduced by 50%	37 [31–43] (21%)	*67* [*66–69*] *(2%)*	57 [52–60] (9%)	*70* [*68–71*] *(2%)*
1.4	ProbIT (75%)	Probability of indirect transmission via humans reduced by 75%	31 [26–37] (33%)	66 [65–68] (4%)	53 [48–57] (15%)	69 [67–70] (3%)
1.5	MR (1/year)	Movement restrictions based on testing all herds once per year	*47* [*43–53*] *(0%)*	62 [60–64] (10%)	*63* [*60–66*] *(−1%)*	64 [62–65] (10%)
1.6	MR (4/year)	Movement restrictions based on testing all herds four times per year	37 [32–42] (22%)	63 [60–65] (9%)	55 [51–58] (12%)	64 [62–66] (9%)
1.7	EradR (1/year)	Eradication in randomly selected herds	43 [38–49] (8%)	65 [63–67] (6%)	59 [55–62] (6%)	67 [64–68] (6%)
1.8	EradH (1/year)	Risk-based eradication	33 [27–37] (31%)	**57 [54–59] (18%)**	49 [44–52] (22%)	**57 [55–60] (20%)**

The initiation date of control measures is dependent on the respective scenario (early initiation: onset on 1^st^ January 2007, late initiation: onset on 1^st^ January 2010). Scenarios with the lowest (highest) predicted relative reduction are marked *italic* (**bold**) for each scenario.

^*^Results presented in Schulz *et al*.^5^ except results for scenario EradH.

When combining two control measures, the observed reduction rates varied from 0% (Scenario 2.3) to 83% (Scenario 2.24) in the low-prevalence scenario (Table 3). With the exception of two scenarios, the predicted relative reduction was smaller in the high-prevalence scenario compared to the low-prevalence scenario. The observed reduction rates varied from 8% (Scenario 2.1) to 65% (Scenario 2.24) in the high-prevalence scenario. Scenarios including risk-based eradication led to the greatest reduction rates.

**Table 3:** Predicted median herd prevalence of the default scenarios run without any control measures (first row) and relative reductions observed for the combinations of two control measures six years after initiation of control (early initiation: predictions on 31^st^ December 2012, late initiation: predictions on 31^st^ December 2015).

Scenario ID	Scenario acronym		Predicted median herd prevalence in %* [90% prediction interval] (relative reduction)			
			Early initiation of control		Late initiation of control	
			Low-prevalence scenario	High-prevalence scenario	Low-prevalence scenario	High-prevalence scenario
0	**No control**		47 [42–52]	69 [67–71]	62 [59–65]	71 [69–73] (0%)
2.1	AB (50%)	ProbIT (50%)	36 [22–49] (24%)	*64* [*54–73*] *(8%)*	52 [39–64] (16%)	*66* [*55–74*] *(7%)*
2.2		ProbIT (75%)	30 [17–42] (37%)	63 [52–71] (9%)	49 [36–60] (22%)	65 [55–73] (9%)
2.3		MR (1/year)	*47* [*30–61*] *(0%)*	57 [48–65] (17%)	*59* [*48–70*] *(5%)*	58 [48–65] (19%)
2.4		MR (4/year)	35 [22–50] (25%)	59 [48–68] (15%)	51 [38–63] (17%)	61 [50–65] (15%)
2.5		EradR (1/year)	42 [26–56] (10%)	61 [51–70] (11%)	55 [41–67] (12%)	63 [57–67] (12%)
2.6		EradH (1/year)	33 [17–45] (31%)	52 [41–62] (24%)	44 [31–57] (29%)	53 [46–66] (26%)
2.7	AB (100%)	ProbIT (50%)	22 [11–35] (54%)	56 [31–67] (19%)	41 [23–55] (34%)	60 [26–72] (16%)
2.8		ProbIT (75%)	15 [7–26] (68%)	58 [37–63] (16%)	38 [22–50] (40%)	58 [39–69] (19%)
2.9		MR (1/year)	30 [15–46] (36%)	50 [42–59] (27%)	48 [28–63] (22%)	52 [34–58] (27%)
2.10		MR (4/year)	21 [9–33] (56%)	52 [35–59] (25%)	38 [20–50] (39%)	51 [37–62] (28%)
2.11		EradR (1/year)	26 [11–40] (45%)	55 [36–66] (20%)	41 [24–57] (34%)	55 [32–67] (23%)
2.12		EradH (1/year)	15 [6–28] (68%)	44 [12–52] (37%)	29 [11–43] (54%)	40 [20–52] (44%)
2.13	ProbIT (50%)	MR (1/year)	38 [32–43] (19%)	60 [58–62] (13%)	58 [53–61] (8%)	62 [60–64] (13%)
2.14		MR (4/year)	28 [23–33] (41%)	61 [59–63] (12%)	49 [44–53] (22%)	63 [61–65] (12%)
2.15		EradR (1/year)	33 [28–39] (29%)	62 [61–64] (9%)	52 [48–56] (16%)	65 [63–66] (9%)
2.16		EradH (1/year)	23 [19–27] (52%)	53 [51–55] (23%)	40 [34–45] (36%)	52 [50–55] (26%)
2.17	ProbIT (75%)	MR (1/year)	32 [27–39] (31%)	58 [57–60] (15%)	54 [49–58] (14%)	61 [59–63] (15%)
2.18		MR (4/year)	23 [19–29] (50%)	59 [57–59] (14%)	45 [40–49] (28%)	62 [60–64] (13%)
2.19		EradR (1/year)	28 [23–34] (41%)	61 [59–63] (12%)	48 [42–52] (24%)	63 [61–65] (11%)
2.20		EradH (1/year)	18 [15–22] (62%)	50 [48–52] (27%)	34 [29–39] (45%)	49 [46–51] (32%)
2.21	MR (1/year)	EradR (1/year)	29 [25–34] (37%)	57 [55–59] (18%)	46 [42–49] (26%)	58 [56–60] (19%)
2.22		EradH (1/year)	26 [22–30] (46%)	52 [50–54] (24%)	42 [38–46] (33%)	53 [51–55] (26%)
2.23	MR (4/year)	EradR (4/year)	21 [18–25] (55%)	45 [43–47] (35%)	36 [32–40] (42%)	45 [43–47] (37%)
2.24		EradH (4/year)	**8 [7–10] (83%)**	**24 [23–26] (65%)**	**16 [14–20] (74%)**	**21 [18–23] (71%)**

The initiation date of control measures is dependent on the respective scenario (early initiation: onset on 1^st^ January 2007, late initiation: onset on 1^st^ January 2010). Scenario acronyms are explained in the main text and in Table 2. Scenarios with the lowest (greatest) predicted relative reduction are marked *italic* (**bold**) for each scenario.

^*^Results presented in Schulz *et al*.^5^ except scenarios including EradH.

The combination of three control measures increased the maximum reduction rate to 94% (Scenario 3.24, Table 4) in the low-prevalence scenario. In all scenarios, the relative reduction was greater in the low-prevalence scenario compared to the high-prevalence scenario, where the reduction rates ranged from 15% (Scenario 3.3) to 82% (Scenario 3.24, Table 4). Finally, when combining all four control measures (Table 5), the greatest observed reduction rate in the low-prevalence scenario was 97%, while in the high-prevalence scenario it was 88%.

**Table 4:** Predicted median herd prevalence of the default scenarios run without any control measure (first row) and relative reductions observed for the combinations of three control measures six years after initiation of control (early initiation: predictions on 31^st^ December 2012, late initiation: predictions on 31^st^ December 2015).

Scenario ID	Scenario acronym			Predicted median herd prevalence in %* [90% prediction interval] (relative reduction)			
				Early initiation of control		Late initiation of control	
				Low-prevalence scenario	High-prevalence scenario	Low-prevalence scenario	High-prevalence scenario
0	**No control**			47 [42–52]	69 [67–71]	62 [59–65]	71 [69–73] (0%)
3.1	AB (50%)	ProbIT (50%)	MR (1/year)	*36* [*22–49*] *(23%)*	55 [47–63] (20%)	*53* [*40–65*] *(14%)*	56 [47–64] (21%)
3.2			MR (4/year)	26 [16–38] (44%)	56 [45–64] (19%)	44 [32–56] (29%)	57 [47–65] (19%)
3.3			EradR (1/year)	32 [19–46] (31%)	*59* [*48–68*] *(15%)*	48 [34–61] (23%)	*61* [*49–70*] *(14%)*
3.4			EradH (1/year)	21 [11–34] (54%)	50 [40–57] (27%)	36 [23–48] (43%)	46 [34–58] (35%)
3.5		ProbIT (75%)	MR (1/year)	32 [20–43] (33%)	54 [44–61] (22%)	50 [38–61] (20%)	54 [46–63] (24%)
3.6			MR (4/year)	21 [13–33] (55%)	55 [44–64] (21%)	40 [28–50] (36%)	56 [45–65] (22%)
3.7			EradR (1/year)	26 [15–38] (44%)	57 [46–68] (18%)	44 [31–55] (30%)	58 [48–68] (19%)
3.8			EradH (1/year)	16 [8–27] (66%)	43 [36–55] (37%)	29 [19–41] (53%)	42 [33–52] (41%)
3.9		MR (1/year)	EradR (1/year)	29 [18–41] (39%)	53 [43–61] (24%)	42 [32–53] (32%)	55 [45–59] (23%)
3.10			EradH (1/year)	24 [13–36] (48%)	47 [38–57] (31%)	37 [25–49] (40%)	48 [42–57] (33%)
3.11		MR (4/year)	EradR (4/year)	20 [11–31] (57%)	42 [31–50] (40%)	32 [21–42] (48%)	39 [33–51] (45%)
3.12			EradH (4/year)	8 [3–14] (84%)	20 [11–27] (71%)	13 [7–22] (80%)	15 [9–21] (79%)
3.13	AB (100%)	ProbIT (50%)	MR (1/year)	22 [10–35] (53%)	50 [40–56] (27%)	42 [25–56] (32%)	48 [18–55] (32%)
3.14			MR (4/year)	15 [7–25] (68%)	52 [34–60] (28%)	33 [14–44] (48%)	50 [26–61] (30%)
3.15			EradR (1/year)	38 [32–43] (61%)	50 [37–64] (25%)	35 [20–49] (43%)	51 [28–61] (28%)
3.16			EradH (1/year)	11 [4–20] (77%)	51 [11–46] (45%)	22 [10–32] (65%)	34 [12–45] (52%)
3.17		ProbIT (75%)	MR (1/year)	19 [7–30] (59%)	38 [41–54] (30%)	38 [22–49] (39%)	47 [19–55] (34%)
3.18			MR (4/year)	13 [6–22] (72%)	48 [18–58] (31%)	29 [16–39] (53%)	48 [22–58] (33%)
3.19			EradR (1/year)	15 [5–26] (68%)	49 [32–58] (29%)	31 [16–43] (50%)	51 [24–61] (29%)
3.20			EradH (1/year)	8 [3–15] (83%)	36 [27–45] (48%)	18 [9–27] (71%)	31 [12–40] (57%)
3.21		MR (1/year)	EradR (1/year)	16 [8–28] (65%)	46 [33–53] (34%)	30 [15–41] (52%)	45 [28–53] (37%)
3.22			EradH (1/year)	13 [5–22] (72%)	38 [28–47] (44%)	24 [11–34] (61%)	36 [23–45] (50%)
3.23		MR (4/year)	EradR (4/year)	10 [4–18] (79%)	32 [19–43] (53%)	21 [10–31] (67%)	30 [15–40] (58%)
3.24			EradH (4/year)	**3 [1–6] (94%)**	**12 [6–18] (82%)**	**6 [2–10] (91%)**	**6 [2–11] (91%)**
3.25	ProbIT (50%)	MR (1/year)	EradR (1/year)	21 [17–25] (55%)	54 [52–56] (22%)	39 [34–42] (38%)	55 [53–57] (22%)
3.26			EradH (1/year)	18 [14–22] (62%)	48 [46–50] (30%)	34 [29–38] (46%)	48 [46–50] (32%)
3.27		MR (4/year)	EradR (4/year)	15 [12–17] (69%)	41 [39–43] (41%)	29 [25–33] (53%)	40 [38–42] (43%)
3.28			EradH (4/year)	5 [4–6] (90%)	19 [18–21] (72%)	11 [7–13] (83%)	14 [12–15] (81%)
3.29	ProbIT (75%)	MR (1/year)	EradR (1/year)	17 [14–21] (64%)	52 [50–54] (24%)	34 [29–39] (46%)	53 [51–55] (25%)
3.30			EradH (1/year)	14 [11–17] (70%)	46 [44–48] (33%)	29 [24–33] (54%)	45 [43–47] (37%)
3.31		MR (4/year)	EradR (4/year)	12 [9–14] (75%)	39 [37–41] (44%)	25 [21–29] (60%)	37 [35–40] (48%)
3.32			EradH (4/year)	4 [3–5] (92%)	17 [16–18] (76%)	8 [7–10] (87%)	11 [9–12] (85%)

The initiation date of control measures is dependent on the respective scenario (early initiation: onset on 1^st^ January 2007, late initiation: onset on 1^st^ January 2010). Scenario acronyms are explained in the main text and in Table 2. Scenarios with the lowest (greatest) predicted relative reduction are marked *italic* (**bold**) for each scenario.

^*^Results presented by Schulz *et al*.^5^ except scenarios including EradH.

**Table 5:** Predicted median herd prevalence of the default scenarios (first row) and relative reductions observed for the combinations of four control measures six years after initiation of control (early initiation: predictions on 31^st^ December 2012, late initiation: predictions on 31^st^ December 2015).

Scenario ID	Scenario acronym				Predicted median herd prevalence in %* [90% prediction interval] (relative reduction)			
					Early initiation of control		Late initiation of control	
					Low-prevalence scenario	High-prevalence scenario	Low-prevalence scenario	High-prevalence scenario
0	**No control**				47 [42–52]	69 [67–71]	62 [59–65]	71 [69–73] (0%)
4.1	AB (50%)	ProbIT (50%)	MR (1/year)	EradR (1/year)	*20* [*12–30*] *(56%)*	*49* [*41–57*] *(29%)*	*34* [*24–44*] *(45%)*	*50* [*42–57*] *(30%)*
4.2				EradH (1/year)	17 [9–27] (64%)	40 [35–52] (42%)	28 [18–39] (54%)	42 [31–51] (41%)
4.3			MR (4/year)	EradR (4/year)	13 [8–22] (72%)	36 [27–46] (47%)	25 [16–34] (61%)	34 [24–44] (52%)
4.4				EradH (4/year)	4 [2–8] (91%)	14 [10–22] (80%)	8 [4–13] (87%)	10 [5–16] (86%)
4.5	AB (50%)	ProbIT (75%)	MR (1/year)	EradR (1/year)	16 [9–24] (65%)	48 [38–56] (31%)	30 [21–38] (53%)	48 [38–57] (33%)
4.6				EradH (1/year)	13 [7–20] (73%)	41 [32–49] (41%)	25 [15–33] (61%)	38 [28–49] (46%)
4.7			MR (4/year)	EradR (4/year)	11 [6–16] (77%)	35 [27–43] (49%)	21 [13–30] (66%)	33 [23–41] (53%)
4.8				EradH (4/year)	3 [1–6] (93%)	13 [9–19] (81%)	6 [3–10] (91%)	7 [4–13] (90%)
4.9	AB (100%)	ProbIT (50%)	MR (1/year)	EradR (1/year)	11 [5–19] (77%)	43 [29–50] (38%)	23 [14–33] (62%)	41 [27–49] (42%)
4.10				EradH (1/year)	9 [4–15] (82%)	35 [27–43] (50%)	18 [8–27] (71%)	31 [19–39] (56%)
4.11			MR (4/year)	EradR (4/year)	7 [3–13] (86%)	30 [14–37] (57%)	16 [6–24] (74%)	25 [14–33] (64%)
4.12				EradH (4/year)	2 [1–4] (96%)	9 [2–13] (87%)	4 [1–7] (93%)	5 [1–7] (94%)
4.13	AB (100%)	ProbIT (75%)	MR (1/year)	EradR (1/year)	9 [5–15] (80%)	37 [30–46] (46%)	21 [11–29] (66%)	39 [25–46] (45%)
4.14				EradH (1/year)	7 [3–12] (86%)	34 [22–43] (51%)	15 [7–22] (76%)	29 [13–36] (60%)
4.15			MR (4/year)	EradR (4/year)	6 [2–10] (86%)	28 [25–35] (59%)	13 [5–19] (79%)	22 [10–30] (68%)
4.16				EradH (4/year)	**1 [1–3] (97%)**	**9 [6–11] (88%)**	**3 [1–5] (95%)**	**4 [1–5] (95%)**

The initiation date of control measures is dependent on the respective scenario (early initiation: onset on 1^st^ January 2007, late initiation: onset on 1^st^ January 2010). Scenario acronyms are explained in the main text and in Table 2. Scenarios with the lowest (greatest) predicted relative reduction are marked *italic* (**bold**) for each scenario.

^*^Results presented by Schulz *et al*.^5^ except scenarios including EradH.

### Effects of a late initiation of control

The initiation of control measures was simulated on 1^st^ January 2010 (late initiation of control) in both the low- and high-prevalence scenarios.

When initiating control in 2010, the effects of individual control measures are comparable to the effects observed in the early-initiation scenario for both the low- and high-prevalence scenarios (Table 2). However, lower levels were observed in all scenarios with individual control measures, with the exception of a reduction in high-risk antibiotics in 50% of the herds in the low-prevalence scenario. Nevertheless, in the high-prevalence scenario, a later initiation of control led to a similar or a slightly greater predicted relative reduction. When control measures were combined, both the smallest and the greatest relative reduction rates were found in the same scenarios as in the respective low- or high-prevalence scenario with early initiation of control measures (Tables 3–5). However, in contrast to the low-prevalence scenario, in which the relative reduction decreased with a late initiation of control, most relative reduction values in the high-prevalence scenario remained constant or increased slightly in the late-initiation scenario. In all scenarios, the risk-based selection of herds for eradication led to greater reduction rates compared to the randomly selected herds. For all simulated initial prevalence and control initiation scenarios, the greatest reduction was observed in Scenario 4.16, in which all control measures were combined in their most intensive versions. Even in the high-prevalence scenario with late initiation of control measures, a relative reduction of 95% was observed (Table 5) and the 90% prediction interval was similar to the low-prevalence scenario with late initiation of control measures.

## Discussion

A simulation model mimicking the spread and control of LA-MRSA among Danish pig herds between 2006 and 2015 was enhanced by an additional control measure: eradication of LA-MRSA in herds selected based on their risk of spreading LA-MRSA via pig movements. The effects of this risk-based eradication were calculated for four scenarios with different levels of initial prevalence (low-prevalence vs. high-prevalence scenarios) and the date when the control measures were initiated (early- vs. late-initiation scenario). The four potential control measures (reduced use of high-risk antibiotics (in 50% or 100% of the herds), increased biosecurity (by 50% or 75%), movement restrictions (based on testing herds once or four times per year), and eradication (random or risk-based selection of herds) were evaluated individually and combined to investigate their potential for reducing LA-MRSA spread 6 years after their initiation. Almost all control measures showed potential for reducing the LA-MRSA herd prevalence, with greater reduction rates when control measures were combined. Control measures were not sufficient to clear LA-MRSA from all herds within 6 years of initiation in any of the tested scenarios (regarding initial LA-MRSA prevalence and initiation date of control). Risk-based eradication led to greater reduction rates when implemented individually and in all tested control measure combinations, independent of the considered prevalence and initiation scenario.

Eradication of LA-MRSA in positive herds could reduce the predicted herd prevalence. This effect might even increase when eradication is combined with movement restrictions, limiting the risk of re-introduction via pig movements. Only 7.5% of the breeding and multiplier herds and 5% of the other herd types were assumed to start the eradication process, as depopulation and re-stocking of more herds might lead to ethical and economic issues. Selecting herds randomly might result in fewer administrative costs, as risk-based selection would require updated information on the size of the out-going contact chain of each herd that tested positive for LA-MRSA. Given that information on pig movements is available in the Danish pig movement database, and calculating the size of out-going contact chains is reasonably straightforward, the risk-based approach could be relatively easily implemented.

The combination of a reduction in the use of high-risk antimicrobials (and thus of decreased transmission rates) in all herds and increasing biosecurity by 75% led to a reduction of 68% in the low-prevalence scenario with early initiation of control (Scenario 2.8, Table 3). This example also showed that the combination of two control measures that were not based on testing all herds indicated a high reduction potential when implemented at a low herd prevalence. However, in the high-prevalence scenario, only the combination of movement restrictions and risk-based eradication based on testing all herds four times per year led to a comparable reduction rate of 65% with the early initiation of control (71% with late initiation of control) (Scenario 2.24, Table 3) when combining two control measures.

When combining four control measures, all 16 combinations showed reduction rates equal to or above 50% in the low-prevalence scenario with early initiation of control, whereas in the high-prevalence scenario with early initiation of control, 8 out of 16 control scenarios showed reduction rates equal to or above 50%. Both examples highlight the importance of adapting control measures to the current LA-MRSA prevalence in the considered region/country to ensure a successful effect.

Eradication, as well as other control measures, lead to costs for the respective pig herds and it is therefore important to examine the cost-effectiveness of the simulated strategies. This would enable a better evaluation of these control measures, as the effectiveness and the costs of a potential control programme both represent essential aspects of the decision-making process.

The effect of the reduction in the use of high-risk antimicrobials is closely related to the reduced within-herd transmission rates that were assumed in case that a herd terminates the use of tetracyclines and β-lactams^6,16^. Any intervention strategy that would reduce within-herd transmission rates might lead to similar effects.

In the current study, we simulated high initial prevalence scenarios in order to study the effectiveness of control measures under the current high herd prevalence situation in Denmark. In the absence of information regarding future movement data, we used historical movement data for the simulations. If future movement and contact patterns are substantially different from the historical data, then the results of these measures might also be different. Nevertheless, Schultz *et al*.^12^ studied the movement patterns in Danish pig herds over the simulated period of time and showed that the patterns were quite consistent over time, which supports the assumptions in this study.

In this study, we repeated scenarios that have been presented earlier^4^. These scenarios were supplemented with new control measures and a higher level of initial prevalence. We find it important to present all scenarios so that the reader has a full overview of the effectiveness of the control measures and the extra contribution of newly simulated control scenarios, as well as the effect of all scenarios under a high initial prevalence level.

In conclusion, all tested control measures showed potential to reduce the spread of LA-MRSA among Danish pig herds to different degrees. Risk-based eradication led to greater reduction rates compared to random selection of herds during the eradication process. In the high-prevalence scenario, combinations of control measures including risk-based eradication clearly indicated a more considerable reduction potential than the random selection of herds.
